# ANALYSIS OF RISK FACTORS FOR SUCCESS OF LUMBAR SPINAL STENOSIS SURGERY

**DOI:** 10.1590/1413-785220162406161696

**Published:** 2016

**Authors:** Caroline Oliveira Brêtas, Larissa Furbino de Pinho Valentim, Nelson Elias, Igor Machado Cardoso, Joelmar César de Almeida, Charbel Jacob

**Affiliations:** 1. Escola Superior de Ciências, Santa Casa de Misericórdia de Vitória (EMESCAM), Vitória, ES, Brazil.; 2. Santa Casa de Misericórdia de Vitória (HSCMV), Department of Orthopedics and Traumatology, Spine Group, Vitória, ES, Brazil.

**Keywords:** Spinal stenosis, Risk factors, Treatment outcome

## Abstract

**Objective:**

To identify the patient profile that obtains better clinical and quality of life improvement after lumbar spinal stenosis surgery, comparing the results in the pre and postoperative periods.

**Methods:**

Thirty-seven patients with lumbar spine stenosis submitted to surgery were prospectively evaluated. Through the 36-Item Short Form General Health Survey (SF-36) questionnaire we performed a preoperative analysis to identify morbidities and social security benefit earning. The SF-36 is a subjective postoperative questionnaire to assess surgical success six months after the surgery.

**Results:**

There were unfavorable outcomes in patients who received social security benefits and in those who had morbidities. According to the SF-36 score, the surgical result is better when the patient is non-smoker (p=0.05), non-hypertense (p=0.040), non-diabetic (p =0.010) or non sedentary (p=0.019), respectively on mental health, pain, social aspects and general health domains.

**Conclusion:**

The patient profiles that best benefit from the surgery are those who do not have morbidities and had no social security benefit. Evidence Level II, Prospective Study.

## INTRODUCTION

Spinal stenosis results from a channel narrowing which causes compression of the neural structures of the spinal bones and adjacente soft tissue.^1^ It may be classified as primary, caused by congenital or developmental postnatal changes, or secondary resulting from degenerative changes or as a result of infection, trauma or surgery.^2^

Although the precise incidence is unknown, it is estimated that lumbar stenosis affects one in 1,000 patients per year, over the age of 65 years old.^3^ Due to the continuous increase in life expectancy, there is a concomitant increase of this pathology. It occurs most frequently at L4-L5 level, followed by L5-S1 and L3-L4 levels.^4^ Stenosis of degenerative origin is uncommon in patients younger than 50 years, in contrast to the congenital origin.^5^

The natural history of the disease remains poorly understood, with studies reporting that about half the patients remain clinically stable and a quarter of them improved or worsened.^6^

Sciatica pain complaint occurs in up to 95% of cases and neurogenic claudication in up to 91% of the cases. Sensory changes in the lower limbs are present in 70% of patients.^4^

The initial treatment is always conservative. It consists to interrupt activities that trigger the symptoms, relative rest and use of analgesics such as acetaminophen, opioids and nonsteroidal anti-inflammatory drugs for a short period of time.^4^

Surgery is indicated when there is no response to conservative treatment for at least 12 weeks associated with a significant change in performing daily activities, according to the Oswestry Disability Index (ODI) and the 36 Item Short Form General Health Survey (SF-36), besides moderate to severe radicular pain based on the visual analog scale (VAS).^4^

Surgical treatment aims to improve the quality of life of patients, reduce low back and radicular pain and improve the neurological deficit. Regarding patients undergoing surgery, over 80% of them reported symptomatic relief within two years.^4^

The most common indication for surgical treatment of degenerative lumbar pathology is arthrodesis or fusion, which shows good results in approximately 70% of cases in a long term assessment.^7^

The best recommendation for obtaining surgical success is to ensure, firstly, that the surgical indication is accurate, i.e., that the pathology is surgically remediable, and then, one must consider other factors that can influence the expected outcome.

The success of an outcome is probably best predicted considering the predominant objective of the surgery.^8^ Thus, for decompression surgery of a herniated disc or spinal stenosis, the most important result should be to reduce leg pain or sensory disturbances and/or to restore the ability to walk.^6^ Under these conditions, the recovery of normal function of daily activities is also important, although this typically comes with time after the main symptoms have been resolved.^8^

 The definition of quality of life integrates objective and subjective indicators, a wide range of life domains and individual values. It can be categorized into five dimensions: physical well-being, material well-being, social well-being, emotional well-being and development of activities (such as work).^9^

Extended sick leave and financial benefit due to disease are considered consistent risk factors for a poor outcome regarding the return to work.^8^

There is a relationship between obesity, diabetes and lumbar spinal stenosis surgery. In morbidly obese or diabetic patients, there is an increased risk of postoperative infection and poor wound healing. Moreover, obese patients are at increased risk of phlebitis and pulmonary embolism.^10^

Smoking is related to a negative effect on lumbar fusion and patient dissatisfaction with the surgery.^11^

The aim of this study was to demonstrate the patient profile that obtains better surgical success, i.e., clinical improvement coupled with improved quality of life. A better understanding of prognostic factors will enable patients and physicians to develop realistic and individual perspectives about the surgical outcome. 

## MATERIALS AND METHODS

This is a longitudinal prospective study. The study was approved by the Research Ethics Committee on human subjects of *Escola Superior de Ciências da Santa Casa de Misericórdia de Vitória* under registration number 037163/2014. The patients studied were from the Orthopedic outpatient of *Hospital Santa Casa de Misericórdia de Vitória*. We evaluated 37 patients with degenerative lumbar spinal stenosis, which have been submitted to surgical decompression of the spinal canal and circumferential arthrodesis with pedicle screws and lumbar cages according to the level of spinal injury. All participants signed a free and informed consent form and agreed to answer the questionnaires. The questionnaires used in the preoperative period were SF-36, social security benefits and morbidities, conducted through interviews during the patient's admission before undergoing the surgical procedure. Six months after surgery, the same patients were interviewed by phone. At this stage the SF-36 questionnaire and the subjective questionnaire developed by the researchers were employed in order to evaluate the surgical success. Pre- and postoperative evaluations were performed by the same examiners.

The inclusion criteria in this study were: patients aged 18 years old or older presenting preoperatively degenerative stenosis of the lumbar canal, disabling low back pain or sciatica pain refractory to conservative treatment for at least 12 weeks. Exclusion criteria were patients younger than 18 years old without stenosis of the lumbar canal or non degenerative stenosis of the lumbar canal, who did not undergo surgical treatment and those who did not agree with the informed consent form.

All patients were evaluated preoperatively by plain radiographs of the lumbosacral spine in standing position in anteroposterior (AP) and lateral (P) views, and magnetic resonance imaging (MRI) of the lumbar spine in order to identify the disease, confirm the diagnosis and determine the degree of injury.

The quality of life questionnaire SF-36 (Medical Outcomes Study 36 - Item Short-Form Health Survey) consists of 36 questions divided into 11 questions with their respective items that are the basis of calculations for an evaluation of eight components: (1) functional capacity, (2) physical aspects, (3) pain, (4) general status (5) vitality, (6) social aspects, (7) emotional aspects, and (8) mental health. The individual receives a score in each área which ranges from 0 to 100, where zero is the worst score and 100 the best, i.e., the higher the score, the better the wellness and functionality. Each dimension of the questionnaire is separately evaluated.^10^ Secondary earning was assessed by questioning whether the patient was retired or received pension benefit from the National Social Security Institute (INSS). During the interview the patient was also asked about obesity, physical inactivity, alcohol consumption, smoking, menopause, hypertension, diabetes *mellitus*, thyroid disease and rheumatic disease. Obesity was assessed by the body mass index (BMI), which considers obese patients with BMI greater than or equal to 30. Patients were considered sedentary when they did not engage in regular physical activity. The other clinical conditions were considered present when refered by the patients during interviews. No further laboratory tests were performed in addition to image exams and surgical risk assessment.

Surgical success was achieved when through increased score in SF-36 questionnaire or when the patient reported improvement of quality of life, leg pain, back pain, or when the patient regained the ability to walk or return to previous work activities. In order to characterize the profile of patients undergoing surgery from the results, frequency measurements and percentages for qualitative variables (gender, pension earning, morbidities and surgical success) were used. The comparison of the percentage gain in the postoperative values ​​in relation to the preoperative period of each domain of the SF-36 of each morbidity was performed by the Mann-Whitney U test for independent samples. The significance level was 5% (0.050). Statistical analyzes were performed using SPSS (Statistical Package for Social Sciences) version 23.

A qualitative study with postoperative data was carried out analysing the following parameters: improvement of leg and spine pain after surgery, ability to deambulate, return to work activities and choice to operate if they would have known the surgical outcome. The most frequently observed morbidities were correlated to the presence or absence of secondary earning according to the questionnaire responses.

## RESULTS

Thirty seven patients were interviewed in the preoperative period; the sample included 16 men (43.2%) and 21 women (56.8%). The mean age was 53±14 years old. During the six months follow-up period one patient died. A sample of 36 patients was left for postoperative comparison. The investigated morbidities are shown in Table 1.

**Table 1:** Caracteristics of the sample population according to the variables gender, morbidities and social security earning. Vitória, ES, Brazil, 2016.

Variables	n	%
Gender		
Female	21	56.80%
Male	16	43.20%
Social security benefit		
Yes	21	56.80%
No	16	43.20%
Obesity		
Yes	9	24.30%
No	28	43.20%
Smoking		
Yes	9	24.30%
No	28	43.20%
Hipertension		
Yes	18	46.80%
No	19	53.20%
Diabetes *mellitus*		
Yes	5	13.50%
No	32	86.50%
Sedentary lifestyle		
Yes	21	56.80%
No	16	43.20%
Alcoholism		
Yes	1	2.70%
No	36	97.30%
Thyroid disease		
Yes	4	10.80%
No	33	89.20%
Rheumatic disease		
Yes	8	21.60%
No	31	78.40%
Menopause		
Yes	12	57.10%
No	9	42.90%

**Figure 1:**
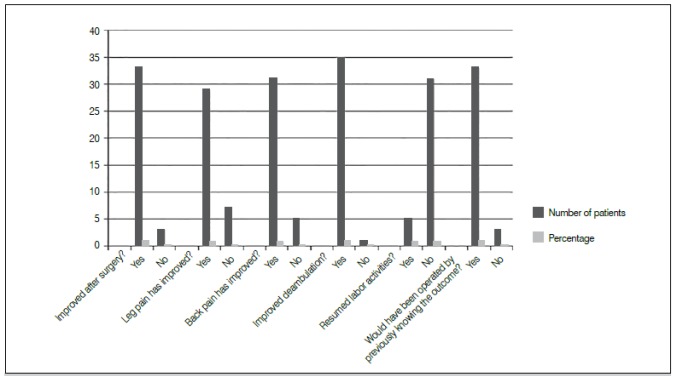
Surgical outcome after six months follow-up. Vitória, ES, Brazil, 2016.

The results showed that most patients achieved surgical success according to evaluation by the subjective questionnaire, as shown in Figure 1. In contrast, the variable returning to work activities showed a slight improvement of 13.9%. Most of patients receiving social security benefits did not take over their previous working activities (only 14.2% retook work activities), however, this fact was not due to the lack of improvement postoperatively, but instead to receiving social security benefits that supplemented their income or job function changes.

Regarding patients who did not obtain leg or spine pain relief, improved ambulation or showed regret for having been operated, morbidities related to this group were hypertension, sedentary lifestyle, smoking, diabetes and obesity. (Table 2)

**Table 2:** Surgical outcome after six months follow-up, correlating worse outcome with morbidity and social security benefit. Vitória, ES, Brazil, 2016.

Variables	No improvement of leg pain		No improvement of spine pain		No imprvement of walking ability		No return to working activities		Would have been operated by previously knowing the outcome	
	n	%	n	%	n	%	n	%	n	%
Obesity	3	33.30%	2	22.20%	0	0%	7	77.80%	1	11.10%
Smoking	1	11.10%	1	11.10%	1	11.10%	9	100%	1	11.10%
Hipertension	6	33.30%	4	22.20%	1	5.60%	16	88.90%	3	16.70%
*Diabetes mellitus*	2	40%	1	20%	0	0%	4	80%	1	20%
Sedentary lifestyle	3	14.30%	3	14.30%	0	0%	18	85.70%	2	9.50%
Social security benefit	5	23.80%	3	14.30%	1	4.80%	18	85.70%	3	14.30%

Statistical analysis of data obtained from SF-36 pre- and postoperatively showed that the evaluated domais mental health, general health, social aspects and pain improved after surgery (Table 3) depending on the associated morbidity. Regarding the percentage improvement of SF-36 score, nonsmokers showed a statistically significant improvement in the mental health domain as compared to smokers (*p* = 0.05). Non sedentary patients showed a statistically significant improvement of general health status (*p* = 0.019) and social aspects (*p* = 0.025) as compared to sedentary. Nonhypertense patients showed a statistically significant improvement of the pain domain (*p* = 0.040) as compared to hypertense patients. Non-diabetic patients showed improvement of social aspects domain (p = 0.010), as compared to diabetic patients. 

**Table 3:** Analysis of percentual improvement in each domain of SF-36 according to each morbidity and social security benefit. Vitória, ES, Brasil, 2016.

	Non smoker	Non hypertense	Non diabetic	Non sedentary
	p - value			
Functional capacity	0.312	0.389	0.161	0.505
Physical aspects	0.693	0.791	0.448	0.924
Pain	0.233	0.040*	1.000	0.427
General status	0.368	0.181	0.657	0.019*
Vitality	0.450	0.542	0.657	0.102
Social aspects	0.860	0.443	0.010*	0.025*
Emotional aspects	0.494	0.839	0.134	0.409
Mental health	0.050*	0.938	0.859	0.770

Mann-Whitney U test of independente samples. Statistical significance level 0.05, *p<0.05

## DISCUSSION

Failure of spinal surgery is a problem that has become relevant, justifying its restricted indication, with failure rates ranging from 5 to 50%, as estimated by studies.^12^ The patients' expectations regarding surgery is an important fact to be assessed, since it correlates to patient's satisfaction over the surgery.

In this study we used SF-36 to assess quality of life due to its applicability. Yee et al., ^13^ in a similar study, analyzed this indicator preoperatively and postoperatively with SF-36 and the Oswestry Disability Index (ODI), which showed that in male patients a better general health score in SF-36 and lower limitation due to physical aspects score are better predictors of better expectation regarding decompression surgery in six months follow-up. Moreover, patients with high expectations also showed greater improvements in the domain limitation by the physical aspects in SF-36 after surgery; and the expectations were achieved in 81% of pacientes.^13^ Patients with lower scores in the domains general health, vitality, mental health in preoperative SF-36 did not achieve their expectations regading surgery.^13^ In our study, an important result was a statistically significant improvement of the domains mental health and general health.

We observed a subjective improvement of leg and spine pain in patiets who did not receive social security benefits, as compared to those who received. Moreover, most patients receiving social security benefits did not retake working activities after surgery. This fact had been previously shown in a meta-analysis study by Moraes et al.,^14^ which showed a negative influence that the workers' compensation benefits plays in the outcome of patients undergoing orthopedic and trauma surgery. According to this study, patients with financial compensation undergoing surgery, are twice as likely to get poor results as compared to non-compensated patients.

Regarding the analysis of impact that certain comorbidities have on the improvement of quality of life, our study showed that obesity, hypertension, sedentary lifestyle, smoking and diabetes *mellitus* were more prevalent conditions in patients who did not obtain improvement in leg and spine pain. Regarding this group, there were two contrasting data: the pain symptoms were mostly observed in most non-sedentary patients (26.66%) and nonsmokers (18.51%), as compared to sedentary patients (14.28%) and smokers (11.11%). We attributed this unfavorable finding to the small size of the sample and the short follow-up period of just six months.

The literature reports better ability to walk, better health, higher income, fewer comorbidities and pronounced stenosis are predictors of better subjective outcome,^15^ while depression, increased cardiovascular risk, disorder that affects the ability to walk and scoliosis are predictors of worst subjective outcome.^15^ Being male and younger are predictors of better post-operatory ability to walk.^15^

A study by Andersen et al. ^11^ corroborates our findings. According to the authors, smokers have a negative overall satisfaction regarding surgery, despite the functional outcome measured by the Dallas Pain Questionnaire (Dallas Pain Questionnaire) did not show to influence the outcome. Moreover, smoking doubles the risk of non-union in arthrodesis.^11^ Sandén et al.^16^ have shown that smokers present poorer quality of life, less improvement after surgery and increased use of analgesics during the two year follow-up.

The relationship between obesity and lumbar spinal stenosis surgery was studied earlier by Knuttsson et al., ^17^ whose results showed that obese patients used more analgesics, had increased leg and spine pain, lower quality of life, greater degree of dissatisfaction and worse outcomes of the surgery during two year follow-up.

Forty-one diabetic patients were compared to 124 non-diabetic patients in the study by Takahashi et al.^18^ The final visual analogue scale scores for back pain were higher in diabetic patients than non-diabetic ones (29.3 *vs*. 17.9, *p* = 0.013).^18^ Improvement of leg or back pain was also lower in diabetic patients in this study (40% and 20%, respectively, versus 19.31% and 12.90% for diabetics).^18^

Most studies report a complication rate lower than 10%.^19^ In our study there was one death (2.7%) due to the surgery complication during follow-up caused by pulmonary thromboembolism.

Taylor et al.^20^ reported higher reoperation rates among patients receiving pension compensation (18%), as compared to patients who did not receive social benefit compensations (10%), also higher in patients under 60 years old. Our study did not followed-up enough time to measure the reoperation incidence. 

## CONCLUSION

Surgical success in lumbar spinal stenosis treatment was best observed in patients who did not refer obesity, sedentary lifestyle, high blood pressure, diabetes *mellitus*, smoking or earning social security benefits.
